# A rare case of stent graft-related endoleak during follow-up of aortic arch aneurysm F/B TEVAR

**DOI:** 10.1093/jscr/rjaf419

**Published:** 2025-06-23

**Authors:** Miao Li, Chen Liu, Zhao Liu

**Affiliations:** Department of Vascular Surgery, Nanjing Drum Tower Hospital, The Affiliated Hospital of Nanjing University Medical School, #321 Zhongshan Road, Nanjing 210008, China; Department of Vascular Surgery, Nanjing Drum Tower Hospital, The Affiliated Hospital of Nanjing University Medical School, #321 Zhongshan Road, Nanjing 210008, China; Department of Vascular Surgery, Nanjing Drum Tower Hospital, The Affiliated Hospital of Nanjing University Medical School, #321 Zhongshan Road, Nanjing 210008, China

**Keywords:** aortic arch aneurysm, 3D printing technology, F/B TEVAR, endoleak, blood in phlegm

## Abstract

A 68-year-old male patient was hospitalized with a giant arch aneurysm. Using 3D printing guidance, the aneurysm was successfully sealed, but post-surgery, he developed severe complications. He remained in the intensive care unit for 52 days for observation and was discharged for rehabilitation. During follow-up, recurrent blood in sputum prompted a computed tomography scan revealing intracavitary leakage. After multiple treatments and assessments, it was determined that type IV endoleak resulted from the proximal bare stent graft puncturing the membranous portion of the aortic stent. Subsequent stent graft procedures and precise embolization successfully closed the leakage, resolving the symptoms.

## Introduction

True aortic arch aneurysms are extremely challenging to treat. Traditional open surgery is high-risk and difficult due to the involvement of all major arch branches, making conventional endovascular repair impossible. The advent of Fenestration/Branch Thoracic Endovascular Aortic Aneurysm Repair (F/B TEVAR) offers a minimally invasive approach, presenting a safer and less traumatic alternative for treating true thoracic aortic aneurysms.

## Case report

**Figure 1:**
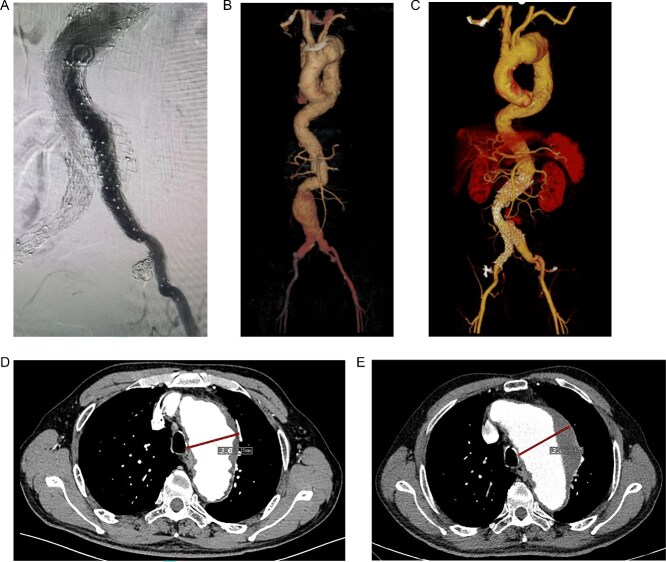
(A) Internal iliac artery embolization and stent graft implantation were performed. (B) Preoperative aortic CTA in 2019. (C) Type Ib endoleak from left iliac artery stent graft. (D) CTA shows the diameter of aortic arch aneurysm was 61.15 mm in July 2019. (E) CTA shows the diameter of aortic arch aneurysm was 72.64 mm in July 2022.

A 68-year-old male was admitted on July 14, 2023, with “intermittent blood in phlegm for over a month.” He had a history of EVAR and underwent right internal iliac aneurysm embolization (Fig. 1A) on July 18, 2019 for an abdominal aortic aneurysm (Fig. 1B). On July 1, 2022, he underwent left iliac artery stent graft implantation due to abdominal pain from a type Ib endoleak (see Fig. 1C). Computed tomography angiography (CTA) findings demonstrated that the maximum diameter of the thoracic aortic aneurysm had reached 72.64 mm and showed a 11.49 mm increase in aortic arch aneurysms over 3 years, which had been reached the established criteria for surgical intervention according to the 2022 ACC/AHA Guideline for the Diagnosis and Management of Aortic Disease [1] (Fig. 1D and E).

**Figure 2:**
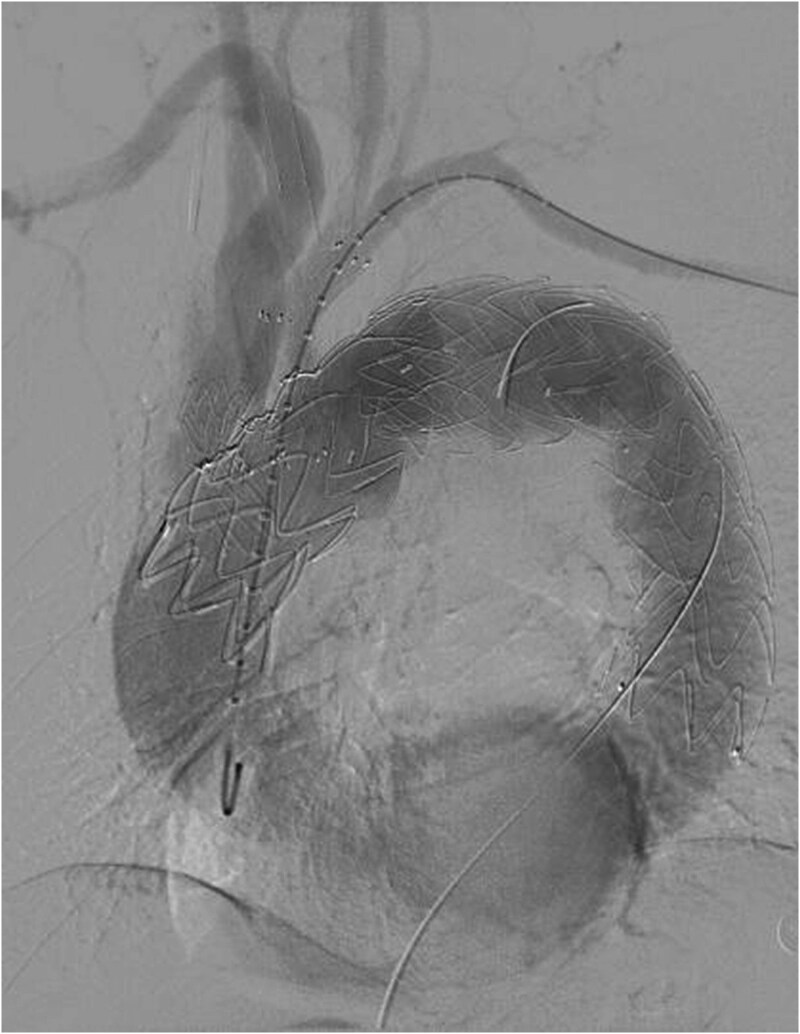
Reconstruction of the three branches of the arch (F/B TEVAR).

The patient underwent F/B TEVAR on August 17, 2022. Physician-modified stent-graft (PMSG) were deployed in all three branches of the arch. Aortic stent graft from Captivia, Medtronic, USA were utilized; Branch stent graft included iliac artery stent graft and coated stent graft, from Medtronic, BD, etc. Following stent graft placement, angiography revealed unobstructed flow in the three branches of the superior arch, and the arterial aneurysm disappeared completely (see Fig. 2). Due to the twisted aorta, the length of the aortic stent graft delivery system was insufficient, leading to the use of a common iliac artery-artificial vessel approach. The procedure was complicated by anastomosis rupture and massive hemorrhage, requiring a 1000 ml blood transfusion. Post-surgery, the patient was admitted to the intensive care unit and developed a high fever, significant hemoglobin drop, and unstable circulation. An urgent computed tomography revealed a retroperitoneal hematoma, which was removed on August 19, 2022. Intraoperative exploration uncovered a large number of blood clots, totaling ⁓1000 ml, with no obvious bleeding points found. The patient developed bacteremia, pulmonary infection, septic shock, acute kidney injury (KDIGO3 stage), and fungal infection. After long-term supportive treatment, the patient was transferred to a general ward on October 10, 2022, and discharged on October 28, 2022.

**Figure 3:**
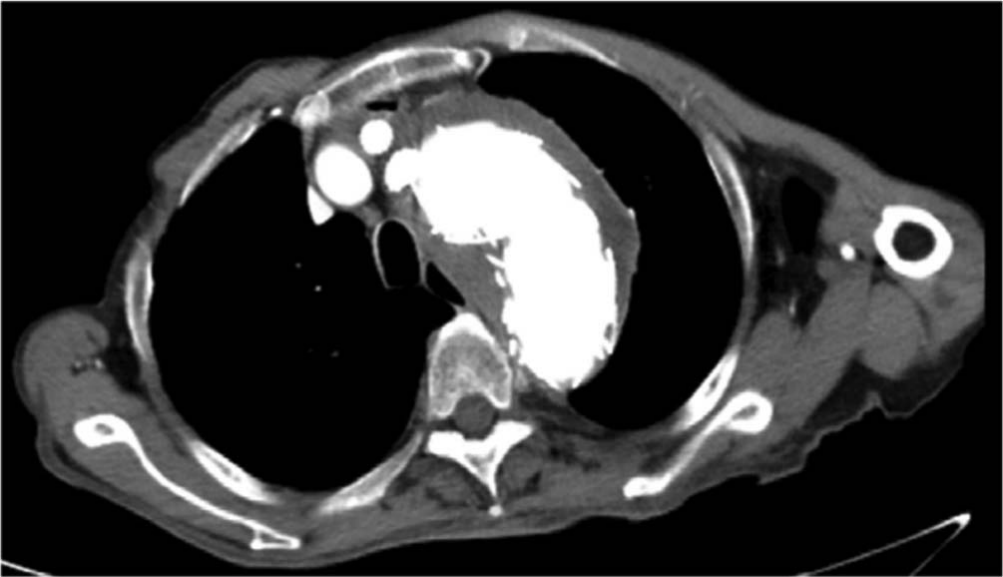
No endoleak was observed upon reexamination in May 2023.

**Figure 4:**
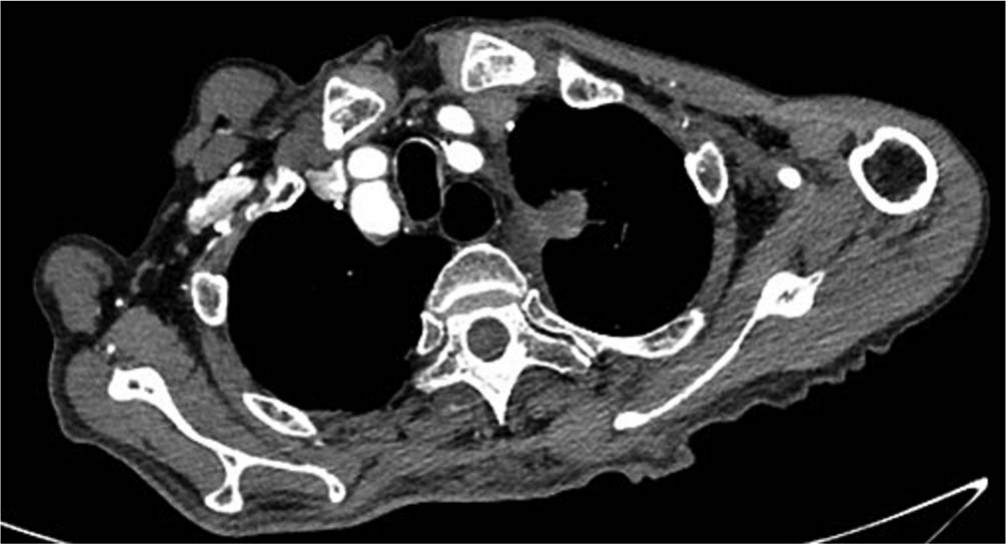
Lung CT results revealed no lung lesions associated with blood in sputum.

**Figure 5:**
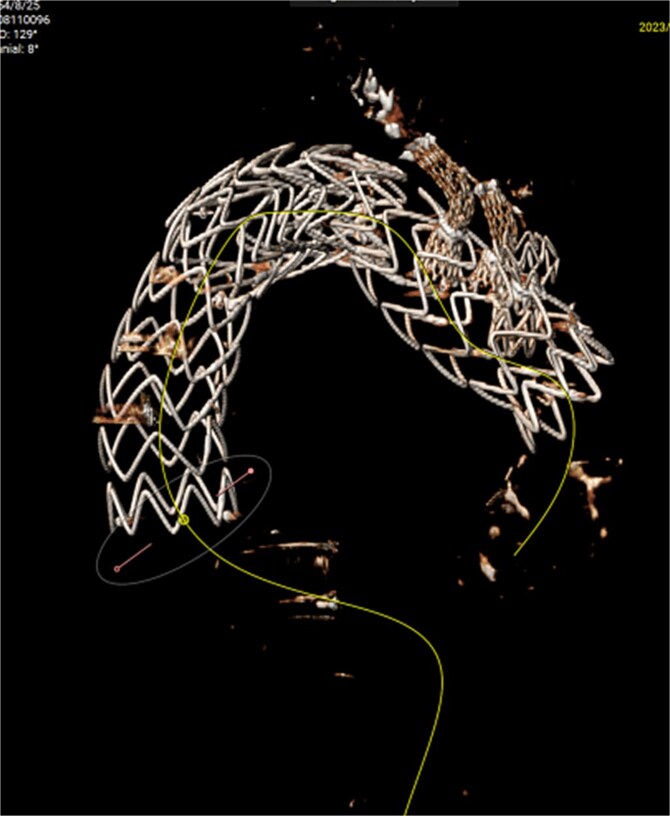
In July 2023, endoleak was observed upon reexamination, possibly of stent graft origin.

**Figure 6:**
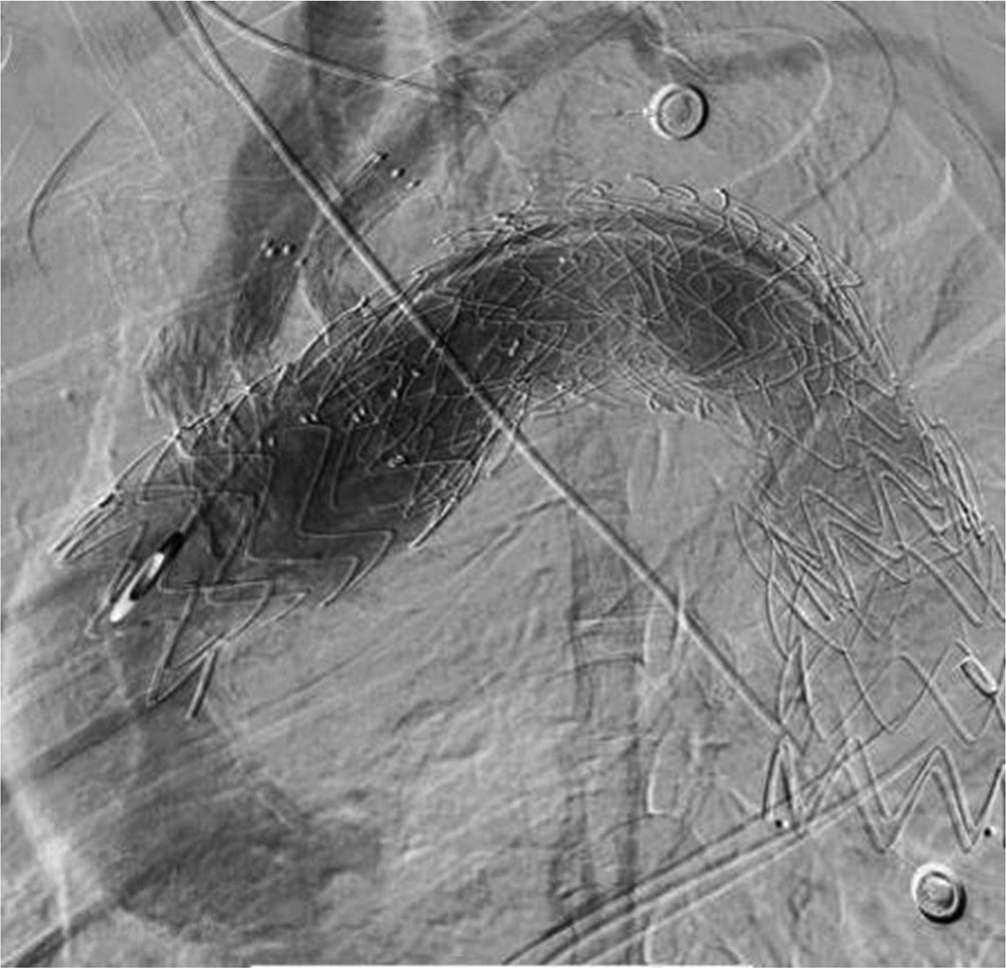
The thoracic aorta was re-implanted with a covered stent graft to cover the breach.

Despite recovery, the patient remained physically exhausted and required ongoing rehabilitation. In May 2023, after a COVID-19 infection, the patient experienced blood in phlegm. CTA confirmed successful aneurysm repair with no endoleak (Fig. 3). Thus the patient was hospitalized in the respiratory department. Relevant pulmonary examination indicators and lung CT results revealed no lung lesions associated with blood in sputum (Fig. 4). However, throughout July, 2023, intermittent blood in phlegm continued, and a CTA on July 19, 2023, revealed a type IV endoleak (Fig. 5). Conversely, aortography on July 27, 2023, showed no obvious endoleak. Closed drainage of the left thoracic cavity was performed, but blood in phlegm recurred on August 9, 2023. A Gore C-TAG stent graft was deployed in the thoracic aortic arch, with its proximal end anchored at the ostium of the left subclavian artery stent graft to ensure complete coverage of the endoleak site both proximally and distally(Fig. 6). Post-surgery, blood in phlegm symptoms abated.

**Figure 7:**
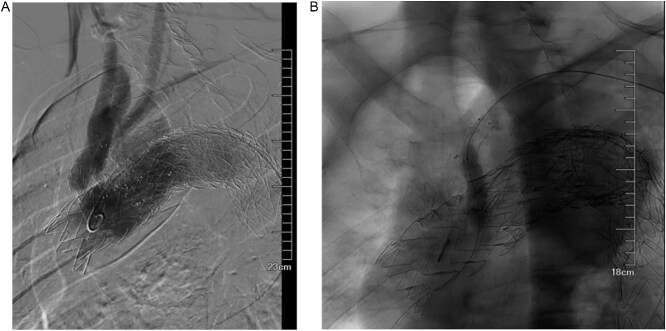
(A) It was hypothesized that the gore aortic stent graft compressed the original subclavian artery BD stent graft, causing a type Ic endoleak. (B) Left subclavian artery stent graft implantation and balloon dilation on December 4, 2023, improved the endoleak.

In November 2023, the patient had another recurrence of blood in the phlegm, and a CTA at an external hospital revealed extravasation around the thoracic aortic stent graft. And endoluminal embolization of the left bronchial artery was performed. Upon admission on December 1, 2023, symptoms had improved. It was hypothesized that the Gore aortic stent graft compressed the original subclavian artery BD stent graft, causing a type Ic endoleak (Fig. 7A). Left subclavian artery stent graft implantation and balloon dilation on December 4, 2023, improved the endoleak (Fig. 7B). However, the patient was readmitted on December 29, 2023, due to recurrent blood in phlegm, leading to transcatheter embolization of the right bronchial artery on January 3, 2024. Symptoms improved significantly, and the patient was discharged.

**Figure 8:**
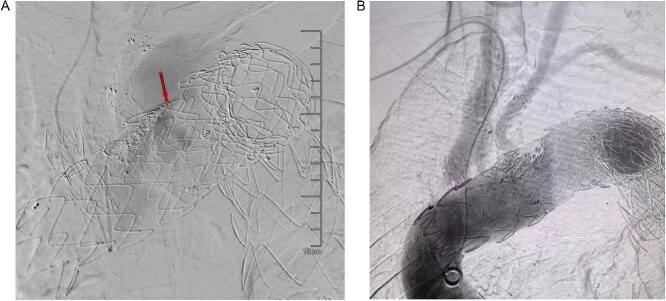
(A) On March 1, 2024, the angiography showed endoleak (marked by arrows) at overlapping part of stent graft. (B) Dense embolization was performed on the residual breach of the stent graft membrane.

On March 1, 2024, the patient was readmitted for recurrent blood in phlegm. CTA revealed a type IV endoleak caused by membrane rupture. Angiography identified the endoleak at the stent graft junction (Fig. 8A). Controllable embolization coils were deployed to embolize the rupture. Post-surgery, symptoms disappeared (Fig. 8B).

## Discussion

True aortic arch aneurysms are challenging, traditionally requiring complex open surgery [2, 3]. F/B TEVAR offers a minimally invasive alternative with high safety and efficacy but demands technical proficiency to avoid complications [4]. In this case, 3D printing facilitated precise stent graft placement, yet the patient’s complex condition led to severe complications, including massive hemorrhage, infections, and multiple endoleaks.

We utilized 3D technology for enhanced accuracy [5] and created a model of the patient’s aorta. Compared to traditional methods, 3D printing allows for precise positioning without cerebral protection, reducing surgical duration and enhancing success rates. However, disease complexity can still lead to serious complications [6].

Despite successful endovascular repair, the patient suffered from underlying diseases, prolonged operation, trauma, and significant intraoperative blood loss. Postoperatively, complications included high fever, decreased hemoglobin, retroperitoneal hematoma, sepsis, pulmonary infection, septic shock, acute kidney injury (KDIGO stage 3), and coagulation disorders.

The patient’s twisted aorta, extending from the arch to the iliac arteries, measured up to 850 mm. We selected the Captivia stent graft with an 830 mm delivery sheath and employed a retroperitoneal approach. Despite precautions, severe arterial calcification led to anastomotic rupture and substantial intraoperative bleeding, requiring prompt hemostasis with over 2000 ml blood loss.

Due to the patient’s medical history, pressor drugs were used despite inadequate blood volume, and insufficient intraoperative transfusion and delayed replenishment of blood and coagulation factors caused rapid thrombosis, microcirculation contraction, and coagulation dysfunction [7]. Despite hematoma clearance, kidney failure and lung infection persisted. Blood cultures revealed bacterial and viral infections, requiring repeated anti-infection treatments. After ventilator weaning, intermittent fever persisted, likely triggered by the inflammatory response to stent graft implantation, surgery, and anesthesia [8]. The patient’s condition improved, but persistent graft-related blood in phlegm followed COVID-19 recovery. A follow-up CTA detected a type IV endoleak (attributed to structural characteristics of the proximal bare stent graft) requiring further embolization after successful sealing with a Gore C-Tag stent graft.

While the patient’s condition and blood in phlegm improved, the direct correlation with the endoleak remains unclear. Several reasons support this conclusion: (i) The onset of blood in phlegm preceded the occurrence of the endoleak, and the volume of blood was relatively small, ˂100 ml per day. Typically, aortotracheal fistulas from endoleaks cause significant blood in phlegm. (ii) Endoleaks can lead to the dilation of cystic aneurysms, which may cause rupture of the pulmonary arterioles, resulting in blood in phlegm. Additionally, due to lacunar enlargement and uncontrolled hypertension, the patient may be at risk of hemorrhagic shock due to rupture of the pleural aneurysmal sac. However, the patient did not develop acute hemorrhagic shock or other severe conditions. Further investigation is required to ascertain the relationship between the endoleak and the occurrence of blood in the phlegm. In rare instances, endoleak may evade detection until an aneurysm rupture occurs [9].

F/B TEVAR while providing many advantages, it still poses significant surgical risks. In addition to common complications such as endoleak, complications caused by intracavitary repair of giant aneurysms can lead to serious consequences. Additionally, the use of various complex graft combinations can also result in unpredictable complications. Employing reasonable treatment strategies, intraoperative monitoring, close observation, timely management of complications, and postoperative follow-up are crucial throughout the treatment process.
